# PReS-FINAL-2131: Hunting for biomarkers in juvenile dermatomyositis

**DOI:** 10.1186/1546-0096-11-S2-P144

**Published:** 2013-12-05

**Authors:** F Bellutti Enders, A Van Royen-Kerkhof, B Prakken, F Van Wijk, R Scholman, W De Jager

**Affiliations:** 1 Department of Pediatric Immunology and Rheumatology, Lausanne University Hospital, Lausanne, Switzerland; 2 Pediatric Immunology, Medical University Utrecht, Utrecht, Netherlands

## Introduction

Juvenile Dermatomyositis (JDM) is a systemic autoimmune disorder in which the immune system targets the microvasculature of skeletal muscles, skin and other organs, with for the most part an unknown immunopathogenesis. Moreover, evaluation of disease activity remains challenging in juvenile dermatomyositis as muscle enzyme levels and inflammatory markers, routinely used in clinics, are no reliable biomarkers in JDM, especially for monitoring the disease.

## Objectives

To identify a panel of mediators specially related to the inflammatory process in JDM, which might help in clinical assessment and in guiding treatment.

## Methods

We performed a multiplex immunoassay and measured plasma levels of 45 inflammation related proteins in patients with four different disease stages determined by their clinical activity and their treatment. Peripheral blood and clinical data were collected in a prospective way from 25 patients diagnosed with JDM. 15 healthy children and 8 patients with non-autoimmune muscle disease served as controls.

## Results

Patients with JDM at time of diagnosis had significantly higher levels of three proteins compared to patients in remission, patients with non-autoimmune muscle disease and healthy-age-matched controls.

## Conclusion

Our results show that these three proteins (which are currently not named due to a pendent patent application) correspond to the activation status during inflammation in JDM and might be instrumental in monitoring disease activity and treatment guiding.

## Disclosure of interest

None declared.

